# Editorial: Conceptual Categories and the Structure of Reality: Theoretical and Empirical Approaches

**DOI:** 10.3389/fpsyg.2017.00601

**Published:** 2017-04-20

**Authors:** Paul M. W. Hackett

**Affiliations:** School of Communication, Emerson CollegeBoston, MA, USA

**Keywords:** categories, ontology, mereology

The process or activity of forming mental categories is both fundamental and necessary to the existence of all living creatures from bi-polar categories such as safe-dangerous, wet-dry, edible-not edible, to higher and more complex forms of categorial discrimination (see, for example, Greggor and Hackett, 2017). The worlds within which sentient creatures live are made sense of through categories. The identification of categories, and the development of categorical understandings, are also important within research.

This special edition of Frontiers in Psychology stands in support of this last claim with papers by researchers from a diverse selection of disciplinary backgrounds. For instance, there are contributions that employ, a range of psychological perspectives; philosophical, personal construct, cognitive, perceptual, neuropsychological, etc. (for recent research in these areas see: Mahon and Caramazza, 2009; Khalidi, 2015). The articles also reflect a spread of approaches from theoretical to applied and demonstrate the ways in which categorization is understood and employed in research from the sciences and social sciences.

Notions of parthood and part-whole interaction are similarly concepts that permeate many academic areas. In this special edition Hackett reviews parthood in his paper, “Facet Theory and the Mapping Sentence As Hermeneutically Consistent Structured Meta-Ontology and Structured Meta-Mereology.” In both of his contributions Hackett proffers a theoretical framework for the design and interpretation of research that uses categories. He places emphasis upon the identification of super-ordinate ontologies that are present within a research domain and the explication of mereological (part to part and part to whole) relationships within these ontologies. This meta-theoretical approach to knowledge development in the social sciences is couched within the rubric of the mapping sentence and facet theory. Hackett argues that by adopting this approach it is possible to achieve hermeneutic consistency with theoretical and empirical validity.

Another contribution that stresses the mereological arrangement of the categories they identify is that by Kuška et al. These authors considered categorization, or the use of categories to generate knowledge, in an applied and particularistic sense, whilst conducting theory-driven research. In their paper “Free Associations Mirroring Self- and World-Related Concepts,” the authors couched their linguistic research within the framework of personal construct psychology, presenting findings from the use of free-association to investigate how reality was construed. They claim their findings indicated that people construe reality through the employment of basic units of meaningful categorization. Their methodology required respondents to offer words that related to the words *world* and *self*. They claim that this procedure accesses, in a relatively direct manner, what they called the basic units of meaningful categorization. They discovered that some categories were expressed as semantic polarities such as nature versus culture. Some of the other verbal category groupings were related to respondent understanding of the words *world* and *self*-whilst others mediated pathways in this category-based network.

The article, “Language or motor: reviewing categorical etiologies of speech sound disorders” by Farquharson, is also concerned with linguistic categories. However, in this instance the research presented addresses speech disorders. With foundations in her category-based consideration of this applied area, Farquharson stresses the need for better conceptualizations of the mechanisms associated with speech sound disorders. In doing this, she hopes that this will lead to an improvement in the diagnosis and treatment of children with these forms of disorder.

Questions regarding methodological issues associated with identifying categories in psychological research, are posed by Nakatsuji et al. They employed a categorically related form of data analysis by analyzing respondents' similarity ratings. This is a less complex form of multidimensional scaling (MDS) which analogously explicates psychological space through analysing pair-wise evaluations. In the study reported, participants completed a sort procedure, arranging cards on the basis of their degree of similarity. The authors rigorously compare their approach to traditional MDS and discovered their results obtained closely resembled those obtained using non-metric MDS. However, they argue that their approach was parsimonious, needing approximately one third of the time to complete. They further claim superior sensitivity for their approach. In conclusion, the authors proposed their category deriving method to save time when conducting research that assesses the similarity of appearance.

Foxall's paper, Metacognitive Control of Categorial Neurobehavioral Decision Systems (CNDS), offers a highly theoretical real-world application of the effects of categories of neural activity. Human decision-making, says Foxall, often involves a person ignoring the future consequences of their decisions and this disregard is dependent upon activity within the limbic and paralimbic regions of the brain relative to activity in the prefrontal cortex. His model depicts the relationship between categorically distinct neurphysioilogical, behavioral and cognitive systems. The degree of balance achieved between these categories results in, he claims, normal or addictive behavior. The author discusses these neural elements and proposes a category-based structure to allow understanding of the effects of CNDS on behavior.

In his review of Oderberg's (2013) book, Classifying Reality, Hackett again proffers the mapping sentence as a declarative tool that, in this instance, may enable understanding of the writing about ontologies by other scholars, which allows the development of a categorical structure for experiences contained in an ontology. A similar perspective is taken by López-Gil et al. in their use of web ontologies to categorically structure reality. Their ontology uses the semantic web language OWL (Web Ontology Language) to represent rich and a complex mereologically associated knowledge of the world. In taking an applied outlook their view depicts online students' emotional, cognitive and motivational state as a web ontology, which interacts with distance or blended educational systems. Their categorical ontology, which they have empirically tested, does not impose a specific way of organizing emotional responses but is able to models reality in association to student affect and motivation.

As can be seen from the above descriptions, the contributions in the special edition represent an eclectic mixture of empirical and theoretical psychological approaches. These have been applied to investigate phenomena that are categorial or have used categories to better understand psychological events. Together, the papers offer insight into contemporary use of category-based knowledge.

## Author contributions

The author confirms being the sole contributor of this work and approved it for publication.

### Conflict of interest statement

The author declares that the research was conducted in the absence of any commercial or financial relationships that could be construed as a potential conflict of interest.
